# Reimagining Core Entrustable Professional Activities for Undergraduate Medical Education in the Era of Artificial Intelligence

**DOI:** 10.2196/50903

**Published:** 2023-12-19

**Authors:** Sarah Marie Jacobs, Neva Nicole Lundy, Saul Barry Issenberg, Latha Chandran

**Affiliations:** 1 Department of Medical Education University of Miami Miller School of Medicine Miami, FL United States

**Keywords:** artificial intelligence, entrustable professional activities, medical education, competency-based education, educational technology, machine learning

## Abstract

The proliferation of generative artificial intelligence (AI) and its extensive potential for integration into many aspects of health care signal a transformational shift within the health care environment. In this context, medical education must evolve to ensure that medical trainees are adequately prepared to navigate the rapidly changing health care landscape. Medical education has moved toward a competency-based education paradigm, leading the Association of American Medical Colleges (AAMC) to define a set of Entrustable Professional Activities (EPAs) as its practical operational framework in undergraduate medical education. The AAMC’s 13 core EPAs for entering residencies have been implemented with varying levels of success across medical schools. In this paper, we critically assess the existing core EPAs in the context of rapid AI integration in medicine. We identify EPAs that require refinement, redefinition, or comprehensive change to align with the emerging trends in health care. Moreover, this perspective proposes a set of “emerging” EPAs, informed by the changing landscape and capabilities presented by generative AI technologies. We provide a practical evaluation of the EPAs, alongside actionable recommendations on how medical education, viewed through the lens of the AAMC EPAs, can adapt and remain relevant amid rapid technological advancements. By leveraging the transformative potential of AI, we can reshape medical education to align with an AI-integrated future of medicine. This approach will help equip future health care professionals with technological competence and adaptive skills to meet the dynamic and evolving demands in health care.

## Introduction

As futurist Eric Hoffer [1] eloquently expressed,

in a time of drastic change, it is the learners who inherit the future. The learned usually find themselves equipped to live in a world that no longer exists.

This statement encapsulates the challenge in medical education that is, preparing learners for a future yet to be fully understood. Leaders across all levels in medical education must grasp the profound changes that technological advancements, such as what artificial intelligence (AI) will bring to training and clinical practice. The rapidly changing, technology-based medical environment necessitates new skills and competencies for future physicians [2]. It is incumbent upon educators to reassess core competencies for excellence in patient care as well as develop inventive ways to impart and assess new and evolving skills.

Competency-based medical education (CBME) was endorsed in 1999 by the Accreditation Council for Graduate Medical Education (ACGME) and the American Board of Medical Specialties to focus on desired educational outcomes and to support a framework that assesses clinical competencies of trainees irrespective of training time [3]. They endorsed 6 core competencies—patient care, medical knowledge, professionalism, interpersonal and communication skills, practice-based learning and improvement, and systems-based practice. Subsequently, it became clear that specific milestones or standards were needed to implement CBME and accurately assess the learner’s performance [4]. In 2013, the Association of American Medical Colleges (AAMC) built upon the ACGME competency framework by defining 13 core Entrustable Professional Activities (EPAs) that outline the skills and competencies that every US medical school graduate should be entrusted with when entering residency training [5].These EPAs link competencies to observable workplace-based units of activities, with each one integrating multiple core competencies and subcompetencies [6]. This created a framework that the faculty can use to assess the performance levels of learners based on direct observation and has become the foundation of the undergraduate medical education (UME) curriculum in many US medical schools [5].

The recent proliferation of generative AI and deep learning systems such as ChatGPT (OpenAI) and GPT4 (Generative Pre-trained Transformer 4; Open AI), however, signifies a tectonic disruption, creating the opportunity to redefine the aspects of medical practice and medical education, ranging from clinical reasoning and diagnostic processes to patient interaction and outcomes [7-9]. This critical appraisal is not about discarding existing models but ensuring that they meet the demands of the world our learners will face postgraduation. As we navigate this period of disruption, the question emerges—how should the EPAs evolve in an AI-influenced world? The journey ahead is not merely about adapting to change but anticipating it. Adding a new competency or tweaking an existing one will not suffice; we must reevaluate the relevance and applicability of each EPA in light of the changes introduced by AI. In this paper, we analyze the existing EPAs through an AI perspective, discuss their evolution, and identify new EPAs with the opportunities and challenges brought by rapid technological advances and their deep integration into health care.

## A Conceptual Framework of EPAs With AI Integration

The impact of generative AI will be multifaceted, changing the tasks that physicians need to accomplish while simultaneously shifting the process, speed, and supervision by which those tasks are learned [8,10-12]. While there are widespread calls to evaluate the potential implications of AI in medical practice and medical education [9,10,13-16], there are no papers, to our knowledge, that directly apply these positions to the current UME frameworks of instruction. Within this context, the AAMC’s EPAs serve as a vital point of reference, delineating the skills and activities expected of a graduating physician and creating areas of emphasis for curricular design. Because the direct impact of AI on these EPAs remains largely unexplored, there is an urgent need to scrutinize these EPAs through the lens of AI-driven evolution. Results from the AAMC’s Core EPAs Pilot Implementation Program found that in the US medical schools studied, not all EPAs are taught and assessed with equal success [17]. As shown in Textbox 1 [17], this analysis characterized the challenges and opportunities of EPAs by separating them into 3 disparate clusters associated with sequentially decreasing success of implementation.

**Association of American Medical Colleges Entrustable Professional Activities for medical students upon entering residency clustered as described in Amiel et al.**

**Cluster 1: Core of the core Entrustable Professional Activities (EPAs)**

- EPA 1: Gather a history and perform a physical examination
- EPA 2: Prioritize a differential diagnosis following a clinical encounter
- EPA 5: Document a clinical encounter in the patient record
- EPA 6: Provide an oral presentation of a clinical encounter
- EPA 7: Form clinical questions and retrieve evidence to advance patient care
- EPA 9: Collaborate as a member of an interprofessional team

**Cluster 2: Advanced EPAs**

- EPA 3: Recommend and interpret common diagnostic and screening tests
- EPA 4: Enter and discuss orders and prescriptions
- EPA 8: Give or receive a patient handover to transition care responsibility

**Cluster 3: Aspirational EPAs**

- EPA 10: Recognize a patient requiring urgent or emergent care and initiate evaluation and management
- EPA 11: Obtain informed consent for tests and procedures
- EPA 12: Perform general procedures of a physician
- EPA 13: Identify system failures and contribute to a culture of safety and improvement

**Cluster 4: Emerging EPAs**

- Detailed further in a later section

Among the 13 EPAs, the pilot group identified 6 EPAs (EPAs 1, 2, 5, 6, 7, and 9) that are taught consistently and assessed efficiently within the existing curricula of participating schools. They titled this group the “Core of the Core.” The second grouping of EPAs 3, 4, and 8 was titled “Advanced,” and it was felt to be the most prominently represented in senior UME curricula. This group was noted to be somewhat difficult to measure because of their sparse integration with the existing UME curricula across the participating institutions. The third cluster, identified as “Aspirational” (EPAs 10-13), appears to be absent or underdeveloped in most of the participating schools’ UME curricula [17]. The pilot team also suggested the existence of a fourth “Emerging” cluster of EPAs (such as telemedicine) that are not originally included in the AAMC’s list but nevertheless becoming increasingly more important to the medical student role. Our analysis expands the discussion regarding emerging EPAs in a later section.

**Figure 1:**
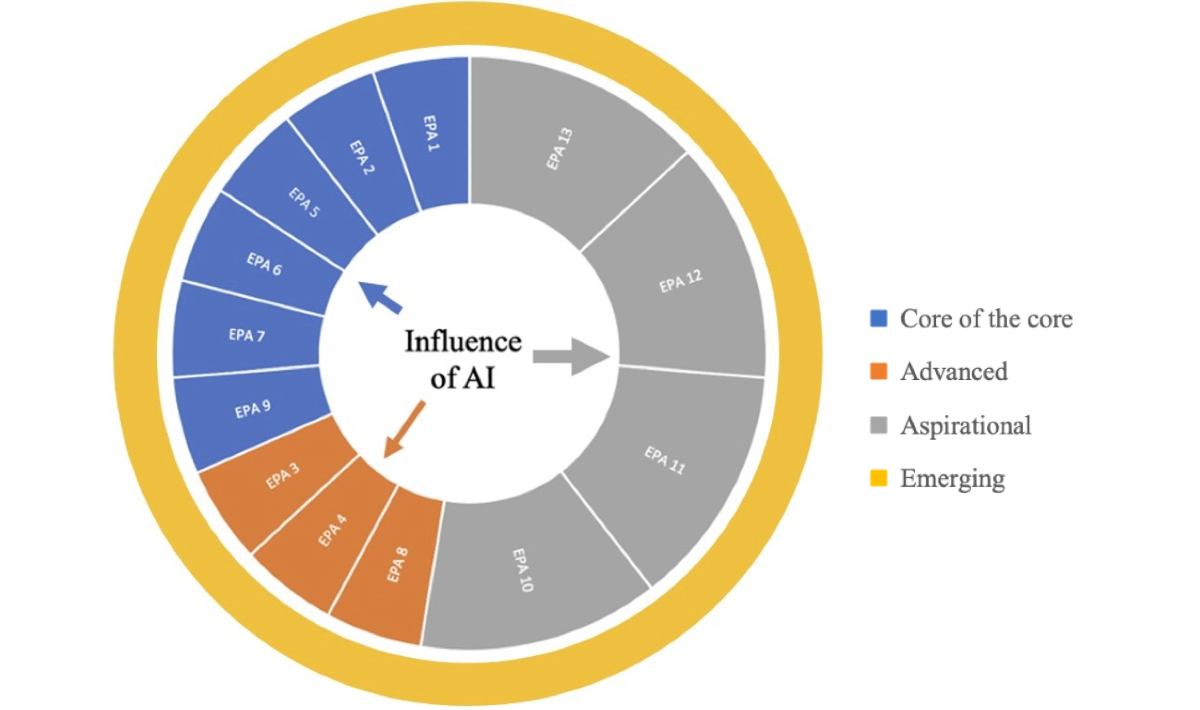
Potential Influences of AI on EPAs. AI: artificial intelligence; EPA: Entrustable Professional Activity.

Using these 4 clusters, we present a conceptual framework to examine the ways AI integration will change and transform the existing landscape. Figure 1 shows the influence of AI across the 3 established clusters of EPAs. The relative size of the 3 portions of the pie represents the degree of influence we believe that AI is likely to have on each of those clusters. For example, we believe that given the ability of generative AI to rapidly classify and transform large sets of data, AI will have the most significant impact on the “Aspirational” cluster of EPAs. Although this cluster is currently underdeveloped in most medical school curricula, AI may create more feasible and functional opportunities for this integration, whereas EPAs in the “Advanced” cluster may be less impacted by AI implementation as they focus on more practical clinical tasks.

In addition, we believe a fourth cluster, termed “Emerging EPAs,” and designated by a ring around the other clusters, will develop and continually evolve in response to the new demands triggered by the integration of technology and AI in health care. While it is impossible to predict the entire list of such emerging EPAs, we propose a few for discussion. Over time, we envision several of these new emerging EPAs becoming part of the “Core of the Core” cluster.

## Analysis of Clusters of EPAs With AI Integration

### Overview

In this section, we explore the potential effects of AI integration on EPAs in the emerging technology-enhanced health care environment. Using a clustered, categorical analysis, we structure the discussion to consider EPAs’ evolution due to AI and assess AI’s far-reaching influence on various physician activities. By investigating the possible synergies and challenges AI presents in each category, we can pinpoint areas where EPAs may require modification, expansion, or redefinition. This aids in initiating a future-focused consideration about how medical education models must adapt for AI-integrated health care. After examining the AI impact on individual EPAs and clusters, we can holistically evaluate the landscape to understand the cumulative effect of these changes on UME.

### Cluster 1: Core of the Core EPAs

The first cluster, “Core of the Core,” identifies EPAs that align well with existing curricula, where learners are provided ample opportunities to practice these skills with direct observation and feedback. These EPAs, such as gathering a history, performing a physical examination, and collaborating as a member of an interprofessional team, form the foundation of medical education. Notably, most surveyed residency program directors expressed confidence that incoming residents could safely perform these skills [18].

While medical students proficiently acquire these skills and medical schools effectively teach them, AI’s emergence could enhance the training and assessment of these core competencies. For instance, AI-based simulation technologies could provide students more opportunities to practice in a controlled environment, and AI-assisted assessment tools could offer more objective and consistent feedback [19]. AI’s influence within this cluster may affect how a task is learned or the pace of mastery. Learning to take a history and perform a physical exam (EPA 1), traditionally necessitating hospital presence or standardized patient interaction, could transform significantly via generative AI. This could allow students to practice these skills at home using avatars and AI chatbots for patient interviews, asking relevant questions and documenting responses. AI also enables immediate, personalized feedback for learners, likely resulting in more efficient and responsive learning [14,20].

However, AI’s integration into medical education’s core areas requires a thoughtful approach, ensuring it enhances, not replaces, the human aspects of these activities. Balancing the practice of core skills on AI-simulated patients versus with direct patient experience is crucial, considering patient presentations seldom align perfectly with textbook descriptions. While the EPAs in this cluster may not need immediate modification, educators must thoughtfully and creatively engage with AI’s potential scope, identifying new assessments to maintain these core skills. Establishing consensus-based guidelines at local and national levels is vital for the appropriate use of generative AI in medical education [17].

### Cluster 2: Advanced EPAs

The second cluster, “Advanced,” encompasses EPAs most prominently represented in senior UME curricula. Activities such as recommending and interpreting common diagnostic and screening tests and giving or receiving a patient handover to transition care responsibility may be insufficiently practiced. Supervision for these activities may lack consistency, which is inadequate for robust evidence collection. AI can mitigate these challenges by providing structured supervision, consistent feedback, and enhancing practice opportunities. AI-powered simulated patients could enable students to practice across diverse clinical scenarios, while AI analytics could identify weaknesses and personalize feedback. AI tools may streamline tasks, suggesting suitable tests based on patient history and symptoms or automating interpretation of imaging studies, pathology slides, electrocardiograms, and laboratory data. These tools could also remind us about pending tasks during a handover.

Similar to cluster 1, AI applications in this cluster aim to augment the teaching process, hasten the learning pace, amplify practice opportunities, and facilitate learner competency assessment. These skills are essential for medical trainees before residency and AI could potentially expedite students’ proficiency and entrustability achievement, enabling time-variable graduation into the health care workforce [20-23].

### Cluster 3: Aspirational EPAs

The third cluster, “Aspirational,” focuses largely on systems-level EPAs that appear to be absent or underdeveloped in the UME curricula of most participating schools. These EPAs include recognizing a patient requiring urgent or emergent care and initiating evaluation and management and obtaining informed consent for tests and procedures. AI could significantly enhance these areas of medical education. For example, AI-driven predictive analytics could assist students in identifying patients needing urgent care [24,25], while AI-powered simulated reality simulations could offer opportunities to practice obtaining informed consent or performing procedures [26].

Moreover, AI could be used to analyze system failures and pinpoint improvement opportunities, thus promoting a culture of safety and continuous learning [27]. In the United States, patient data are stored digitally in Health Insurance and Portability and Accountability Act (HIPAA)–compliant electronic medical records, and while medical records are capable of trending individual variables for laboratory tests and vital signs, effective systems to integrate multiple data points and key health trends over time do not currently exist [28]. At present, annually, health care systems collect enormous amounts of patient care data that remain disparate and disconnected from one another [29]. The integration of AI allows practitioners to glean meaningful information and patterns rapidly thus expediting the efficiency and effectiveness of health care systems, which will be beneficial in the learning process and in assuring safe and quality patient care [30].

Similarly, there is a lack of a deep learning system to identify norms and variances of safe patient care practices. Such a system could offer the physician or trainee personalized arrays of learning opportunities to avoid system failures and enhance patient safety and quality of care [23,27,31]. In addition, deep learning programs that use data from medical records can identify cost of care variations [32,33] and provide learning opportunities through customized feedback on individual practices [23]. We believe that the integration of AI into this cluster will move us closer toward realizing the goals of the Institute for Health Care Improvement’s Triple Aim initiative, which focuses on the experience of care, population health, and cost-effectiveness [34].

However, there also are ways that the EPAs in this cluster may need to be redefined. For example, EPA 11 refers to obtaining informed consent for tests and procedures. The communication skills required to elicit information from the patient (as done in EPA 1 when taking a history and physical) or communicate with other health care providers (EPAs 6 and 9) are different than the ability to explain complex medical concepts at a level that patients and family can understand sufficiently to make life-changing medical decisions. Learners will need to understand how AI technologies function and their use in patient care [35,36]. AI technology is widely known to be difficult to explain and understand; AI has long been criticized for inexplicable “black box algorithms,” and calls to demystify these algorithms have created an entire category of explainable AI [37,38]. Understanding AI requires training, especially its impact on care management decisions, patient outcomes discussions, and ethical decision-making [2,39,40]. If physicians and learners find explaining medical complexities challenging in the existing landscape, adding an additional technological layer could further complicate patient and physician communication.

Clinicians already use AI, such as ChatGPT, for more understandable patient explanations of complex diseases [41], while other educators have proposed using AI to analyze and teach communication in health care [42]. However, without proper training, how effectively will learners and clinicians explain an AI-derived prognosis calculator, Food and Drug Administration (FDA)–approved AI health care apps [43], or other more opaque forms of AI implementation? With the rise of AI-powered health applications for patients, physicians must explain these tools. Explaining technology is not new; it is a challenge encountered whenever complex technology integrates into medical care [44]. As this paper underscores, AI presents a dynamic landscape where clinicians need a fundamental understanding of these technologies and communication skills to relay information to patients. This calls for clinical educators’ concerted efforts to ensure that learners build this knowledge during training.

### Cluster 4: Emerging EPAs

#### Overview

The fourth cluster, “Emerging EPAs,” covers potential EPAs that, while undefined, are crucial given health care’s rapid changes and AI’s impending impact. These EPAs represent the evolving demands, expectations, and skills that physicians will need in the changing health care landscape. Future physicians must adeptly use digital tools in documentation support systems, clinical decision support systems, and operational efficiency systems that are already omnipresent in health care and, with deeper integration of AI, set to become even more influential for patient care. In Table 1, we suggest several new EPAs for further exploration and refinement by the educational leadership community and include several real-life examples illustrating how AI will be applied.

**Table 1:** Emerging Entrustable Professional Activities (EPAs) as a result of artificial intelligence (AI) integration into health care.

Core	Description	Explanation	Examples (current and potential)
14	Proficient use of health care technologies	The medical graduate should be able to use current and emerging technologies effectively and responsibly in health care, including electronic health records, telemedicine platforms, and AI-driven diagnostic tools, to enhance patient care and improve health outcomes.	Wearables and smart devices will collect patient data in real time, and AI will analyze these data for signs or trends of deterioration or complications. Medical students can learn to respond to these automated alerts, intervening early and improving outcomes. AI tools will analyze patients’ medical history, genetics, and lifestyle to predict risks for diseases like diabetes or cardiovascular diseases. Medical students will use these insights to recommend preventative measures. Apps use AI for visual image and radiographic interpretation, and for diagnostic and therapeutic aids. Medical students can use these to confirm their diagnoses and understand the characteristics that the AI deemed significant [45].
15	Understanding and applying health informatics	The medical graduate should be able to understand and apply principles of health informatics, including data management, data privacy, and the use of data for quality improvement and research.	Faculty will use AI applications to populate simulations with realistic patient data that will enable learners to practice using these systems, extracting relevant data, and making clinical decisions based on the information available. AI applications will simulate various cybersecurity threats to a mock health database, allowing students to recognize vulnerabilities and apply best practices for data privacy. AI can generate scenarios where students need to weigh the advantages of data use against potential privacy concerns, thus honing their ethical judgment. Students will explain to patients how AI tools work, what their results mean, and address any concerns or misconceptions patients might have about AI in their care.
16	Demonstrating team skills for transdisciplinary interactions in health care settings	The medical graduate should be able to effectively collaborate within transdisciplinary health care teams, which include other health care professionals as well as experts from other disciplines such as data scientists and bioinformatics specialists.	Medical students will be able to participate in virtual reality simulations where they interact with AI-driven avatars representing different health care disciplines (nurses, pharmacists, physical therapists, etc). This practice will enable them to navigate interdisciplinary scenarios, learning to communicate effectively, and collaborate. AI-driven platforms will simulate complex patient cases requiring inputs from multiple specialties [46]. The AI system will provide feedback on the team’s collective decisions, highlighting areas where interdisciplinary collaboration was effective or could be improved. This includes rare, but highly critical events such as mass casualty incident that require close coordination and teamwork among personnel from numerous disciplines [47].
N^a^	To be defined	Educators need to be vigilant about the possibilities offered by AI integration across many segments of health care and how new EPAs will need to become an integral and required component of medical training. This area is emerging and has the potential to continually evolve. Possible new competencies include the ability to ethically manage autonomous AI systems, and environmental data driven health care	As AI systems become more autonomous in decision-making, students will learn skills to ensure that these systems operate within ethical boundaries and can be overridden or understood by human practitioners when necessary. Students will use AI applications to analyze environmental data (pollutants, allergens, etc) to make predictive health assessments and recommendations for patients, local communities, and larger populations, especially in the context of increasing global environmental changes.

^a^Unknown number of future EPA.

#### Proficient Use of Health Care Technologies (EPA 14)

Medical graduates should proficiently and responsibly use both existing and emerging health care technologies, including electronic health records, telemedicine platforms, and AI-driven diagnostic tools, to enhance patient care and health outcomes. The integration of technology in health care is not future speculation, but a current reality. The existing EPAs, while comprehensive, do not specifically address the use of health care technology. With rapid technological advancement, it is crucial that graduates are comfortable with and able to leverage these technologies to improve patient care. Adding “Proficient Use of Health Care Technologies” as an EPA would highlight this essential skill in medical education, offering a clear benchmark for graduates and preparing them to navigate contemporary health care’s digital landscape. It would encourage medical schools to include technology training in curricula and create standardized skill sets, ensuring future physicians can use these tools effectively.

Considering the constant evolution of technology, mere proficiency in current technologies is not enough for graduates. They must be prepared for continuous learning and integration of emerging technologies throughout their careers. This requires developing a quick adaptability to new technologies and understanding how to incorporate them into practice. Essentially, “learning to learn” becomes a core skill that must be taught in medical education [48]. By incorporating “Proficient Use of Health Care technologies” as an EPA, we highlight not just the importance of technological proficiency at graduation but also the ongoing commitment to learning and adaptation crucial for the ever-evolving health care technology landscape.

#### Understanding and Applying Health Informatics (EPA 15)

Medical graduates should understand and apply health informatics principles, including data management, privacy, and use of data for quality improvement and research. Health care is increasingly data driven, with vast amounts of health data generated for various purposes, from clinical decision-making to quality improvement and research [44]. Consequently, comprehending and applying health informatics principles are essential skills for medical graduates, encompassing both technical issues, such as data management and analysis, and understanding data governance, privacy, and ethical health data use. There is increasing agreement within the literature on this need as shown in multiple recently published papers suggesting various educational methods and frameworks to accomplish this [39,44].

Integrating health informatics into a new EPA acknowledges health care’s increasingly data-centric nature and promotes data science skills’ importance and standardization within medical education. This EPA goes beyond mere technical proficiency, urging learners to understand how data can enhance patient care and health outcomes. This includes data’s potential for leading quality improvement initiatives and informing research, as well as the ethical and legal implications of health data use. As health informatics principles’ understanding and application become critical, this EPA emphasizes the need for medical graduates to be prepared for this new reality and equipped to leverage data to improve patient care.

#### Demonstrating Team Skills for Transdisciplinary Interactions in Health Care Settings (EPA 16)

As health care evolves to become increasingly complex and specialized, the ability to function effectively within transdisciplinary teams will emerge as a critical skill for medical graduates. This includes not only collaboration with other health care professionals but also with experts from diverse disciplines who contribute indirectly yet significantly to care provision. Such experts include data scientists, bioinformatics experts, health informatics specialists, and others whose expertise will be indispensable in ensuring that care is safe, precise, and of high quality. Current EPAs do not explicitly address the need for transdisciplinary team skills, yet the evolving nature of health care teams makes this a critical competency. Medical graduates need the ability to communicate effectively with a broad spectrum of professionals; understand their roles, expertise, and contributions; and collaborate with them to deliver optimal care for patients [25,42]. This extends beyond simply understanding the language and perspectives of other disciplines; it involves appreciating their contributions and integrating their expertise into patient care. This also goes beyond simply communicating with other frontline health care providers as described in EPA 9 “Collaborating on an interprofessional team.” Informatics experts and other similar professionals will play a pivotal role in interpreting health data, developing predictive models, and informing clinical decision-making. This EPA underscores the transdisciplinary future of medicine and ensures that medical students acquire the skills they need to become effective clinicians in a data-driven future.

#### Additional Emerging EPAs

The preceding discussion introduces 3 ideas as emerging EPAs. However, the cluster of these emerging EPAs is yet to be fully delineated. We anticipate rapid growth, evolution, and transformation of the EPAs required for successful health care practices commensurate with the advent of new technologies. Given the accelerated pace of technological evolution in the present era, it is challenging to envisage the nature of novel tools and devices that will shape future health care delivery and the consequent evolution of EPAs. Nevertheless, it is incumbent upon health professions educators to remain vigilant about the obligation to train and assess learners based on these evolving requirements of future physicians. It is essential to ensure that the curriculum offers comprehensive and meticulously designed opportunities for learners to develop proficiency and entrustability in these critical new areas and develop robust self-learning skills prior to them joining the physician workforce.

### Evolution of EPA Clusters With AI Integration

The “Emerging EPAs” cluster will impact all other clusters as the EPAs developed within this group eventually become part of the other 3. The evolution of this cluster will heavily depend on the development of deep learning and AI, as well as the array of tools and applications that arise in the future.

**Figure 2:**
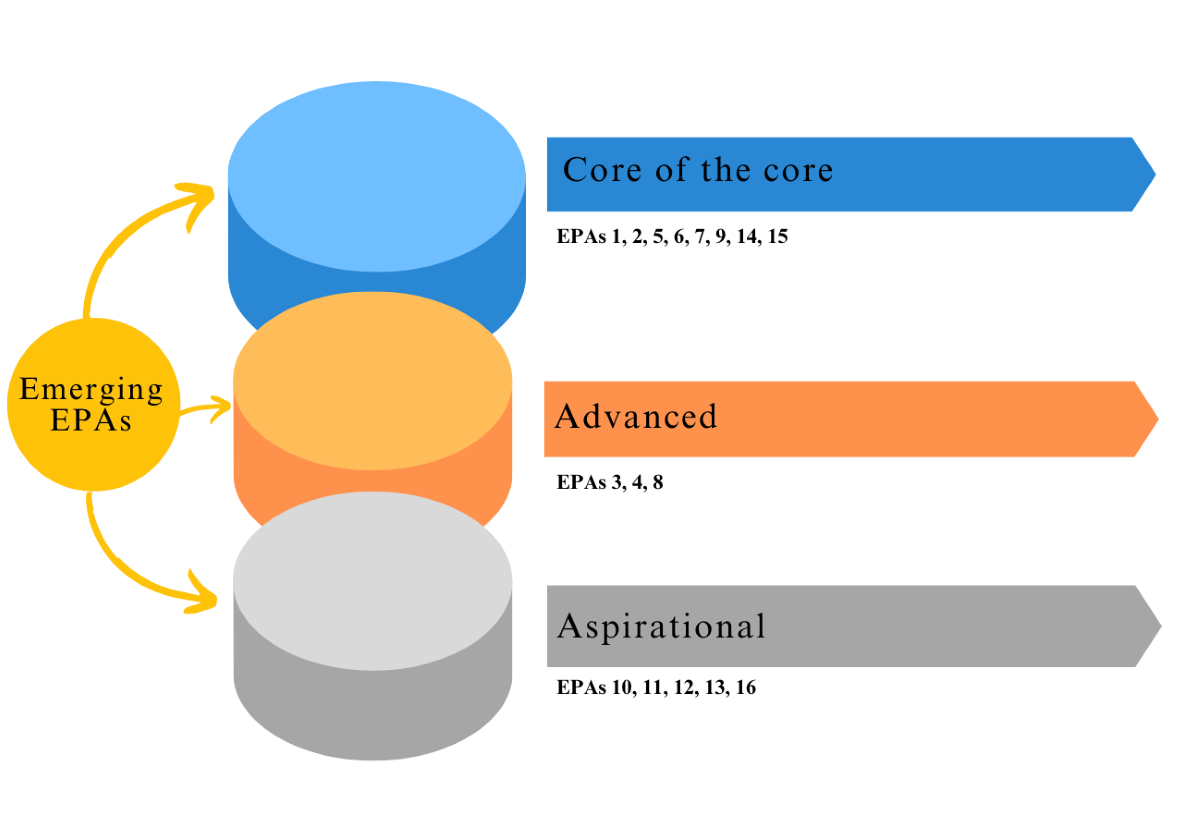
Integration of emerging EPAs into different EPA clusters. EPAs 14, 15, and 16 are EPAs that are proposed in this paper. EPA: Entrustable Professional Activity.

Figure 2 illustrates the potential impact of swiftly integrating AI into health care and health education, demonstrating how it could differentially transform the original 3 clusters of EPAs. AI may affect the EPAs themselves or it may alter the groupings of each cluster, shifting EPAs from one to another by changing the process of medical education. For example, an EPA might move from “Advanced” to “Core of the Core” as new technologies enable medical education to more effectively teach and evaluate previously “Advanced” EPAs. The left circle is the cluster of emerging EPAs. We also propose that the 3 discussed emerging EPAs will move to be part of the existing clusters. As shown in the figure, it is very likely that EPA 14 and 15 will become part of the “Core of the Core” cluster while EPA 16 will become part of the aspirational cluster. These are only conceptual suggestions as it is impossible to predict the number and types of EPAs that will emerge with the advent of more advanced technologies and their integration into health care activities in the future.

## Opportunities and Challenges in the Horizon

We find ourselves at a pivotal juncture in the history of medical education. The pandemic has expedited the adoption of emerging technologies among higher education leaders at an unprecedented rate. Following the announcement of ChatGPT as an open-source large language model trained transformer, its use in various education settings has been widely recognized by faculty and students alike. Simultaneously, policy changes have swiftly adapted to the use of AI in authorship and journal publications [19]. It is incumbent upon educational leaders to consider the broad implications of these transformative forces on the complete spectrum of health education, and proactively steer its trajectory.

As early as 2018, the American Medical Association (AMA) recommended incorporating AI training in medical education [49]. However, a recent literature review reflects a slow response by educators to meaningfully address the role of AI in medicine and its implementation in training [35]. Challenges to the implementation of AI competencies include a lack of standardization of AI definitions, a lack of faculty expertise, and the absence of accreditation guidelines on AI in medical education [7]. The rapid evolution and technological complexity of AI applications add to the difficulty of this task. However, clearly, a basic understanding of AI technology and the opportunities and limitations of its use in health care will be indispensable for future health care providers. There have been some recent attempts to create frameworks for medical school implementation of AI technologies. Krive et al [39] created an AI-driven medical education module, concentrating on 3 competencies: students as evaluators through critical appraisal of AI systems, students as interpreters of AI output, and students as communicators of AI results and processes. Weidener et al [2] evaluated German medical students and concluded that students must possess fundamental knowledge of AI systems, the ability to interpret AI input and output, and comprehension of the appropriate application of AI systems. Our discussion enhances these perspectives by attempting to integrate these competencies with specific EPAs that can be evaluated as discrete skills.

The integration of deep learning and AI tools into health care has the potential to revolutionize the way care is delivered. Many have argued the current structure of health care systems detracts from the fundamental purpose of the physician’s role. Ideally, AI could function as a lever to refocus physicians’ time and attention back to the patient. Instead of being consumed by data analysis and technical tasks, physicians can concentrate more on understanding their patients as individuals, emphasizing the humanistic side of medicine that AI cannot supplant [18]. While many express concerns about the impact of AI proliferation in health care, it is also possible to view it optimistically as having the potential to create space for reflection, partnership, and meaningful patient advocacy at the individual and community levels. It may enable physicians to invest more time in building relationships with their patients, understanding their perspectives, and advocating for their needs. It also provides opportunities for reflection and learning, as physicians can use the insights generated by AI to improve their practice and contribute to the advancement of medicine. These are paradigm-shifting opportunities that could fundamentally change the nature of health care and significantly improve patient outcomes.

In addition to revolutionizing the delivery of health care and the core knowledge that learners must acquire, AI, including generative AI, has the exciting potential to transform the process of medical education. Echoing the narrative surrounding precision medicine, AI introduced the prospect of precision medical education, an approach that is tailored specifically to each learner. This transformation is facilitated by technological platforms capable of delivering immediate, personalized feedback. Projects such as this are currently being piloted at New York University as part of the AMA’s Reimagining Residency Grant where AI programs analyze all resident clinical notes and provide feedback, educational resources, and other suggestions based on their patient panel [50]. Residents receive daily messages with suggested readings based on the differential diagnoses that they encountered the day before. Instant feedback such as this provides the mechanism and the accelerant to clear one of the remaining hurdles to competency-based education, appropriate evaluation, and assessment. With AI-integrated, medical-education platforms, it becomes possible to generate and analyze far more data to confidently answer the fundamental question of whether a learner has adequately achieved their needed competencies. These graduate medical education pilots will pave the way for similar types of integration into UME clinical education, but this is still only 1 sliver of the possibilities where AI may be integrated.

Given these rapidly evolving and exciting changes, it is imperative that leadership organizations responsible for developing guidelines and policies related to medical education focus on this issue urgently. The AAMC has demonstrated leadership in developing the core EPAs for CBME, as well as played a crucial role in developing diversity, equity, and inclusion competencies across the learning continuum [51]. Now is the time for the AAMC to reimagine the role of EPAs in the new era of AI-integrated health care with thought leadership from its membership and groups of engaged educators and leaders. The Accreditation Council on Graduate Medical Education must facilitate the development of specialty-specific milestones related to specific new competencies that will become critical success factors for physicians practicing in the future. In addition to promoting grassroots advocacy from medical students through specific resolutions, in the last decade, the AMA has played a pivotal leadership role in promoting significant innovation in medical education through its Accelerating Change in Medical Education Grant program and has built a national consortium—the AMA ChangeMedEd Consortium [52]. Internationally, the Association of Medical Education in Europe can be pioneer in developing guides focused on the best evidence in this emerging and important facet of medical education [53].

The integration of AI into medical education is also accompanied by potential challenges and ethical dilemmas. A primary challenge lies in the pedagogical adaptation required; educators must find ways to convey complex AI concepts to a predominantly clinical audience, ensuring that future physicians can both understand and critically evaluate the AI tools. This is further complicated by the dynamic nature of AI, where rapid technological advancements can abruptly render current knowledge obsolete, demanding constant curriculum updates. Within AI tools, there is also the challenge of transparency and accountability with many AI algorithms, particularly deep learning models, operating as “black boxes” [54]. For students and faculty, this opacity can be problematic, as they may accept AI recommendations without understanding the underlying rationale, potentially leading to clinical misjudgments. Furthermore, despite its capacity to propose differential diagnoses and management plans, overreliance on AI could compromise the thoroughness of critical thinking and leave students ill-prepared for situations where AI is unavailable or inaccurately applied [55-57]. Students need the knowledge and ability to recognize these situations and respond appropriately.

Ethically, the use of AI in medical education brings forth concerns about data integrity and bias. The inherent biases within the databases used for training large language models have the potential to perpetuate and even exacerbate existing health care inequities and when used in the educational context, they risk introducing skewed perspectives into clinical training [58]. Furthermore, patient privacy is a paramount ethical concern as vast amounts of health data are collected and used in AI tools, often without the patients being aware. As AI is brought further into educational tools, there is concern for the amount of data being collected about students and student data privacy, as the owners of the tools rather than the educational institutions become the owners of the data [59]. The application of AI in assessment should also be approached with caution because of the limited data available on its accuracy and consistency. These limitations carry significant implications for evaluating students and, consequently, for ensuring their ability to make clinical decisions [60]. It is imperative that health care practitioners and health care educators who use these tools are aware of such risks and can actively mitigate these when using such technology [61].

The full integration of AI into health care is not solely about harnessing the power of technology, but about reimagining the role of the physician, the nature of patient care, and the process by which we educate our health trainees. Health professions educators have a vital responsibility at this critical juncture where technology becomes an integral part of daily practice—to regularly evaluate the need for new skills and competencies necessary for future physicians and to shape the transformation of our training systems to ensure opportunities for deliberate and safe practice of such skills. Academic institutions have the further responsibility to reorient toward an “education for life” model, in response to the emerging technologies that serve as powerful catalysts for change [18]. Only then can we state confidently that our graduates can be entrusted to safely care for patients in the emerging world of a symbiotic and seamless relationship between AI and health care. It is imperative that we assume this vital responsibility seriously and immediately for the sake of our patients and our communities who depend on us to do exactly that.

## Abbreviations

AAMC: Association of American Medical Colleges
ACGME: Accreditation Council for Graduate Medical Education
AI: artificial intelligence
AMA: American Medical Association
CBME: competency-based medical education
EPA: Entrustable Professional Activity
FDA: Food and Drug Administration
GPT4: Generative Pre-trained Transformer 4
HIPAA: Health Insurance and Portability and Accountability Act
UME: undergraduate medical education
